# Dothideomycetes and Leotiomycetes sterile mycelia isolated from the Italian seagrass *Posidonia oceanica* based on rDNA data

**DOI:** 10.1186/2193-1801-3-508

**Published:** 2014-09-09

**Authors:** Giorgio Gnavi, Enrico Ercole, Luigi Panno, Alfredo Vizzini, Giovanna C Varese

**Affiliations:** Mycotheca Universitatis Taurinensis (M.U.T.), Department of Life Sciences and Systems Biology (DBIOS), University of Turin, Viale Mattioli, 25, 10125 Turin, Italy; Systematic Mycology Lab, Department of Life Sciences and Systems Biology (DBIOS), University of Turin, Viale P.A. Mattioli, 25, 10125 Turin, Italy

**Keywords:** Dothideomycetes, Fungal molecular phylogeny, Leotiomycetes, Marine fungi, *Posidonia oceanica*, Sterile mycelia

## Abstract

Marine fungi represent a group of organisms extremely important from an ecological and biotechnological point of view, but often still neglected. In this work, an in-depth analysis on the systematic and the phylogenetic position of 21 sterile mycelia, isolated from *Posidonia oceanica,* was performed.

The molecular (ITS and LSU sequences) analysis showed that several of them are putative new species belonging to three orders in the Ascomycota phylum: Pleosporales, Capnodiales and Helotiales. Phylogenetic analyses were performed using Bayesian Inference and Maximum Likelihood approaches.

Seven sterile mycelia belong to the genera firstly reported from marine environments.

The bioinformatic analysis allowed to identify five sterile mycelia at species level and nine at genus level. Some of the analyzed sterile mycelia could belong to new lineages of marine fungi.

## Background

The oceans host a vast biodiversity. Most of the marine microbial biodiversity has not yet been discovered and characterized, both taxonomically and biochemically. Marine fungal strains have been obtained from virtually every possible marine habitat, including inorganic matter, marine microbial communities, seagrasses, algae, driftwood, invertebrates and vertebrates (Imhoff et al. 2011; Rateb and Ebel 2011). However, it is worth mentioning that the fraction of cultivable isolates is very low, around 1% or less, with regard to the overall estimated biodiversity (Rateb and Ebel 2011), similar to the situation with bacteria (Alain and Querellou 2009). Only recently, the immense diversity of microbes in the marine environments has attracted the attention of the scientific community for their almost untouched capacity to produce bioactive natural products (Imhoff et al. 2011). Marine bacteria and fungi produce structurally unique secondary metabolites that often display promising biological and pharmacological properties (Rateb and Ebel 2011) and the remarkably high hit rates of marine compounds in screening for drug leads makes the search in marine organisms quite attractive.

In our previous work (Panno et al. 2013) the diversity, the ecological role and the potential biotechnological applications of marine fungi associated with different parts (leaves, rhizomes, roots and matte) of the seagrass *Posidonia oceanica* (L.) Delile, were investigated. The results showed that the mycobiota associated to young *P. oceanica* plants is very rich, both in term of load and number of species, and is higher than those found on algae, corals, sponges and other seagrasses such as *Thalassia testudinum* Banks ex König and *Zostera marina* L. (Meyers et al. 1965; Newell 1981; Toledo-Hernández et al. 2007; Suryanarayanan 2012; Zuccaro et al. 2008). The mycobiota composition and structure changes significantly in the four parts of *P. oceanica*, displaying a “district specificity” that may be due to multiple factors: specific environmental parameters (nutrients, light, different hydrodynamic motions, etc.), presence of different antagonistic macro- and microorganisms, presence of high concentration of tannic acid in the leaves (Mazzella and Alberte 1986; Pergent et al. 2008). Surprisingly, most of the few fungi that have been already reported associated with *P. oceanica* by other authors (Jones 1963; Cuomo et al. 1985; Garzoli 2013) were not found in our survey. This result could be explained considering the different samplings periods (young plants in spring *vs* old or senescent plants in autumn and winter) in relation to the life cycle of this seagrass, the different parts of the plant analysed, and the focus on marine obligate species or on the total mycobiota.

Moreover the results showed that about 30% of the isolated fungal taxa grow only as sterile mycelia (SM) exclusively associated to matte and rhizomes. Most of these SM remained unidentified, since their ITS sequences displayed low homologies with those present in public databases. On the other hand, this preliminary molecular analysis showed that they could belong to Dothideomycetes O.E. Erikss. and Winka (mainly Pleosporales Luttr. ex M.E. Barr) and Leotiomycetes O.E. Erikss. and Winka (Helotiales Nannf. ex Korf and Lizon).

The Dothideomycetes represents the largest class of Ascomycota Caval.-Sm. and displays a high level of ecological diversity (Hyde et al. 2013; Egidi et al. 2014). They are often found as pathogens, infecting a broad range of hosts (Crous et al. 2013; Hyde et al. 2013), but also as endophytes or epiphytes on living plants and as saprobes degrading cellulose and other complex carbohydrates in dead or partially digested plant matter, in leaf litter or dung. However, their nutritional modes are not limited to associations with plants; several species are mycobionts in lichens, while others occur as parasites on other fungi or animals (Shearer et al. 2009; Hyde et al. 2013; Perez-Ortega et al. 2014; Schoch et al. 2009). Adaptation to fresh- and salt-water habitats has occurred multiple times within the Dothideomycetes (Shearer et al. 2009; Perez-Ortega et al. 2014). Marine Dothideomycetes are considered mainly intertidal, occurring on a wide range of substrata like mangrove habitat, and sea and marsh grasses, and they usually do not produce an anamorphic state. Species that occur completely submerged are mostly parasites or symbionts of seagrasses or marine algae (Suetrong et al. 2009; Hyde et al. 2013).

As regard to Leotiomycetes, this class includes both non-lichen- and lichen-forming fungi. These species colonize a large variety of habitats, and act as saprobes or form parasitic associations with a wide range of other organisms. Besides parasites and saprobes, the group includes endophytes and symbionts of a wide range of plants (Wang et al. 2006a) and it has recently been signalled also from marine environments (Burgaud et al. 2009; Jones and Pang 2012a, [b]). Moreover, similarly to Dothideomycetes, many helotialean fungi are known only from a teleomorphic stage. Their anamorphs are either undiscovered or it is assumed that they have been lost in the process of evolution (Wang et al. 2006b).

The aim of this work was to investigate the systematic and the phylogenetic position of 21 SM isolated in our previous work (Panno et al. 2013), from rhizomes and matte of *P. oceanica,* using the ITS and LSU (28S) rDNA regions.

## Results and discussion

According to the literature many marine-derived fungi (10–40% of the isolates according to the original substrate) are able to grow only as SM in axenic conditions both on normal and specific media containing seawater (Morrison-Gardiner 2002; Raghukumar 2004; Damare et al. 2006; Panno et al. 2013). The high presence of SM in marine environment supports the hypothesis that many fungi may have evolved, as a system of preferential dispersion, the fragmentation of the hyphae in respect to the production of conidia or spores (Damare et al. 2006). Another explanation could be the composition of the culture media that do not mimic *in situ* conditions limiting fungi to grow and/or sporulate *ex situ*.

The predominance of Ascomycota and, in particular, of fungi belonging to Dothideomycetes and Leotiomycetes classes in marine habitats has been discussed in the literature, and the most important hypothesis is that these fungi have evolved efficient adaptations to the aquatic ecosystem (Prasannarai and Sridhar 2001; Vijaykrishna et al. 2006); it is also possible that those groups of fungi are more readily cultivable compared to other and it could be easily recovered when culture-dependent techniques are applied (Baker et al. 2008).

**Figure 1:**
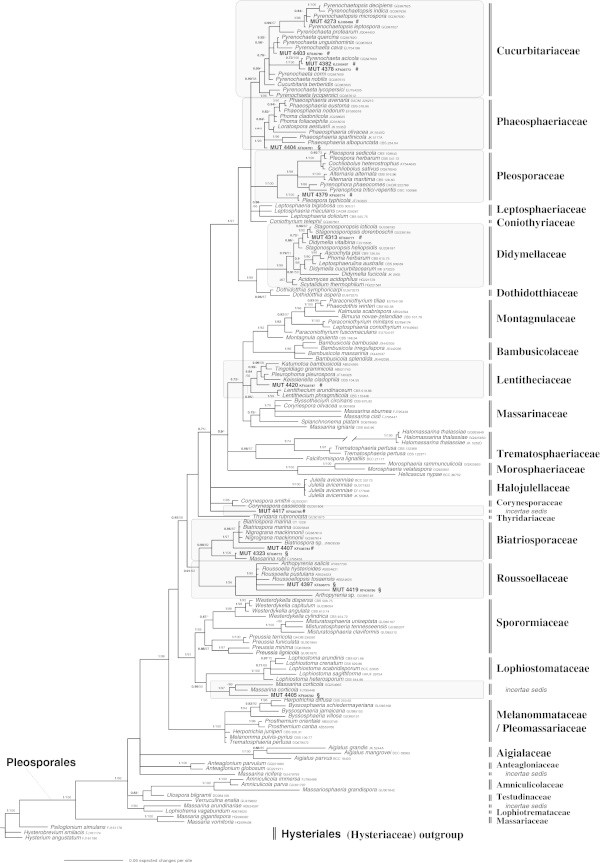
**Bayesian phylogram of Pleosporales (Dothideomycetes) taxa including the 14 fungal isolates (labelled as MUT, in bold), on the dataset of rDNA large subunit (LSU).** Clades designations were based on Hyde et al. (2013). All sequences were from Suetrong et al. (2009), and/or from GenBank. Numbers above branches indicated BPP over 0.70 and MLB over 50. The alignment comprised 1,432 characters and contained 474 variable sites. **#** = strains isolated from *P. oceanica* matte; **§** = strains isolated from *P. oceanica* rhizomes.

**Figure 2:**
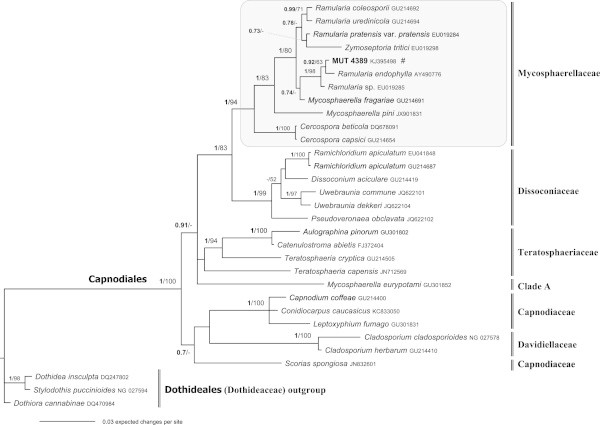
**Bayesian phylogram of Capnodiales (Dothideomycetes) taxa including one fungal isolates (labelled as MUT, in bold), on the dataset of rDNA large subunit (LSU).** Clades designations were based on Hyde et al. (2013). All sequences were from Suetrong et al. (2009), and/or from GenBank. Numbers above branches indicated BPP over 0.70 and MLB over 50. The alignment comprised 1,332 characters and contained 299 variable sites. **#** = strain isolated from *P. oceanica* matte.

**Figure 3:**
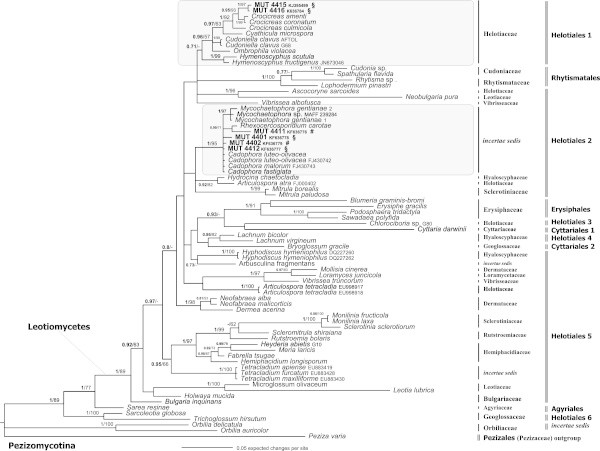
**Bayesian phylogram of Leotiomycetes taxa including the six fungal isolates (labelled as MUT, in bold), on the dataset of rDNA large subunit (LSU).** Clades designations and sequences were from Wang et al. (2006b) and Nekoduka et al. (2010). Numbers above branches indicated BPP over 0.70 and MLB over 50. The alignment comprised 1,065 characters and contains 398 variable sites. **#** = strains isolated from *P. oceanica* matte; **§** = strains isolated from *P. oceanica* rhizomes.

The relevance of SM in marine ecosystems prompted us to analyse the systematic and the phylogenetic position of 21 sterile mycelia, isolated from *Posidonia oceanica.* The ITS and LSU analysis, molecular markers fundamental for systematic and phylogenetic studies, allowed us to attribute the precise taxonomic position: 14 strains belong to Dothideomycetes-Pleosporales, 1 to Dothideomycetes-Capnodiales and six to Leotiomycetes-Helotiales s.l.Both the applied phylogenetic analysis yielded the same topology; therefore, only the Bayesian trees with both Bayesian Posterior Probabilities (BPP) and Maximum Likelihood Bootstrap (MLB) values are shown (Figures 1, 2 and 3). The LSU data matrix of the Dothideomycetes-Pleosporales tree included a total of 159 sequences (14 newly generated, 145 from GenBank). The LSU data matrix of the Dothideomycetes-Capnodiales tree included a total of 31 sequences (1 newly generated, 30 from Genbank). The LSU-5.8S data matrix of the Leotiomycetes-Helotiales tree included a total of 76 sequences (6 newly generated, 70 from GenBank).

### SM of *P. oceanica*in Pleosporales (Dothideomycetes)

The 14 sequences belonging to the Pleosporales-Dothideomycetes were distributed over nine clades corresponding to seven families and two *incertae sedis* clades (Figure 1, Tables 1 and 2).

**Table 1:** **Taxonomic assessment of sterile mycelia isolated from** ***P. oceanica***

MUT code	Part of ***P. oceanica***	Isolated taxa	GenBank accession number	
			ITS	28S
**Dothideomycetes**				
4273	Matte	*Pyrenochaetopsis* sp.	KJ395500	KJ395496
4313	Matte	Didymellaceae sp.	KF636765	KF636771
4323	Rhizomes	*Massarina rubi*	KF636766	KF636772
4378	Matte	*Pyrenochaeta* sp.	KF636767	KF636773
4379	Matte	*Pleospora typhicola*	KF636768	KF636774
4382	Matte	*Pyrenochaeta acicola*	KJ395501	KJ395497
4389	Matte	*Ramularia endophylla*	KJ395494	KJ395498
4397	Rhizomes	Roussoellaceae sp.	KC339235	KF636775
4403	Matte	Cucurbitariaceae sp.	KC339238	KF636780
4404	Rhizomes	Phaeosphaeriaceae sp.	KC339239	KF636781
4405	Rhizomes	*Massarina sp.*	KC339240	KF636782
4407	Matte	*Biatriospora sp.*	KC339242	KF636783
4417	Matte	Pleosporales sp.	KF636769	KF636785
4419	Rhizomes	Roussoellaceae sp.	KC339245	KF636786
4420	Matte	Lentitheciaceae sp.	KF636770	KF636787
**Leotiomycetes**				
4401	Rhizomes	*Cadophora* sp.	KC339236	KF636778
4402	Matte	*Cadophora* sp.	KC339237	KF636779
4411	Matte	*Rhexocercosporidium carotae*	KF636763	KF636776
4412	Rhizomes	*Cadophora* sp.	KF636764	KF636777
4415	Rhizomes	*Crocicreas* sp.	KJ395495	KJ395499
4416	Rhizomes	*Crocicreas* sp.	KF636762	KF636784

**Table 2:** **Sterile mycelia isolated from** ***P. oceanica*** **, MUT Code and percentage of identity with ITS sequence of public database GenBank (NCBI)**

MUT code	Part of ***P. oceanica***	Its sequence homology	% of sequence identity	% of query coverage	GenBank accession number
**Dothideomycetes**					
4273	Matte	*Phoma* sp.	98	100	KF646102
4313	Matte	*Didymella* sp.	99	100	HQ607826
4323	Rhizomes	*Massarina* sp.	96	99	AF383963
4378	Matte	*Pyrenochaeta inflorescentia*	97	91	GU586851
4379	Matte	Pleosporales sp.	100	93	FJ571480
4382	Matte	Phaeophaeria sp.	99	97	EU715675
4389	Matte	*Mycosphaerella punticformis* syn. *Ramularia endophylla*	100	100	EU343240
4397	Rhizomes	Pleosporales sp.	93	99	JN572046
4403	Matte	*Pyrenochaeta* sp.	97	99	KF561892
4404	Rhizomes	*Septoria arundinacea*	100	88	GU361970
4405	Rhizomes	*Lophiostoma* sp.	99	95	HQ914825
4407	Matte	Pleosporales sp.	98	100	HM116750
4417	Matte	Ascomycota sp.	99	96	FJ375144
4419	Rhizomes	Pleosporales sp.	94	100	HM992495
4420	Matte	*Phoma herbarum*	95	85	AB333774
**Leotiomycetes**					
4401	Rhizomes	*Cadophora* sp.	100	95	JF327417
4402	Matte	*Leptodontidium orchidicola*	95	100	GQ302678
4411	Matte	*Rhexocercosporidium* sp.	97	91	DQ249995
4412	Rhizomes	*Cadophora* sp.	94	100	JN859261
4415	Rhizomes	*Crocicreas* cf. *cacal*iae	99	90	FJ005108
4416	Rhizomes	*Crocicreas* cf. cacaliae	99	86	FJ005108

According to Jones et al. (2009), marine Dothideomycetes comprise hundreds of species, the majority belonging to the Pleosporales order, which is the largest one in the Dothideomycetes and comprising a quarter of all dothideomycetous species (Kirk et al. 2008). Species in this order are able to colonize various habitats, and can be epiphytes, endophytes or parasites of living leaves or stems, hyperparasites on fungi or arthropods, lichenized, or saprobes of dead plant stems, leaves or bark (Schoch et al. 2009; Zhang et al. 2009). This order has been extensively investigated in recent years and many new families and marine lineages have been identified. However, although many genera have been sequenced, their phylogenetic relationships remain unresolved and further taxon sampling with a wider range of genes being sequenced is required (Jones and Pang 2012a; Zhang et al. 2012).

According to LSU analysis, four SM isolated from *P. oceanica* matte, MUT 4403, MUT 4378, MUT 4382 and MUT 4273, clearly belong to the family Cucurbitariaceae Winter. MUT 4403, MUT 4378 and MUT 4382 cluster with *Pyrenochaeta* De Not. species; in particular MUT 4382 is strictly related to *Pyrenochaeta acicola* (Moug. and Lév.) Sacc*.* Members of the genus *Pyrenochaeta* are Coelomycetes Grove widely distributed in the environment in soil or in association with wood and plant debris, and several species have been implicated in human infections (de Hoog et al. 2000; Badali et al. 2010); no *Pyrenochaeta* species was so far reported from marine environments (Jones et al. 2009; Abdel-Wahab and Bahkali 2012).

MUT 4273 falls among species of *Pyrenochaetopsis* Gruyter, Aveskamp and Verkley, a Coelomycetes genus closely related to *Pyrenochaeta*. The described *Pyrenochaetopsis* species are all soilborne and mainly associated with gramineous plants (de Gruyter et al. 2010); no species was so far reported as associated to marine environments (Jones et al. 2009; de Gruyter et al. 2010; Abdel-Wahab and Bahkali 2012).

MUT 4404, isolated from rhizomes, clearly belongs to Phaeosphaeriaceae M.E. Barr, (=Clade VII - Phaeosphaeriaceae according to Suetrong et al. 2009), but in an isolated lineage. Future molecular investigations including additional genetic markers will be necessary to better define its taxonomic status. This taxonomic placement is also supported by the ITS sequence analysis; BLAST search indicates a 100% of identity value (88% of query coverage) with a sequence of *Septoria arundinacea* Sacc., a taxon recently included in Phaeosphaeriaceae (Quaedvlieg et al. 2013). Jones et al. (2009) listed 3 genera with marine species in this family: *Carinispora* K.D. Hyde, *Lautitia* S. Schatz and *Phaeosphaeria* I. Miyake, but till now only for 4 species of the latter genus, the LSU sequences are available (*P. albopunctata* (Westend.) Shoemaker and C.E. Babc.*, P. olivacea* Kohlm., Volkm.-Kohlm. and O.E. Erikss., *P. sparticola* Leuchtm*.* and *P. typharum* (Desm.) L. Holm). However, the phylogram does not allow to include MUT 4404 within this genus nor to the close genus *Loratospora* Kohlm. and Volkm.-Kohlm. recently included in this family (Schoch et al. 2009; Suetrong et al. 2009). The latter genus encompasses only one species, *L. aestuarii* Kohlm. and Volkm.-Kohlm. that occurs on *Juncus roemerianus* Scheele culms (Kohlmeyer et al. 1995), a widespread monocot in saline aquatic environments.

MUT 4379, from matte, belongs to the family Pleosporaceae Nitschke. It seems closely related to *Pleospora typhicola* (Cooke) Sacc. (BPP = 1; MLB = 100%), a taxon reported from dead leaves of *Typha* spp. (*Typha* L., Typhaceae Juss.) in moist or wet habitats (Shoemaker and Babcock 1992).

MUT 4313, from matte, belongs to Didymellaceae Gruyter, Aveskamp and Verkley (=Clade IX - Didymellaceae according to Suetrong et al. 2009). More in detail, it seems much closely related to the genus *Stagonosporopsis* Died., which encompasses saprotrophic species from stem and leaves of terrestrial plants (Aveskamp et al. 2010). According to Jones and Pang (2012a) few marine taxa of *Didymellaceae* have been found in this family but only one species, *Didymella fucicola* (G.K. Sutherl.) Kohlm., had been sequenced for LSU marker till now.

MUT 4420, isolated from matte, nests in the Lentitheciaceae Yin. Zhang, C.L. Schoch, J. Fourn., Crous and K.D. Hyde, (=Clade I – according to Suetrong et al. 2009), but occupies an isolated position. Till now only two marine species of Lentitheciaceae, *Lentithecium phragmiticola* (syn. *Massarina phragmiticola* Poon and K.D. Hyde) and *Keissleriella rara* Kohlm., Volkm.-Kohlm. and O.E. Erikss. have been isolated (Jones and Pang 2012a).

MUT 4417, from matte, falls in an isolated position close to *Halojulella* avicenniae (syn. *Julella avicenniae* (Borse) K.D. Hyde) (Halojulellaceae = the Clade X – Julella Fabre according to Suetrong et al. 2009) and related to two *Corynespora* species (*Corynespora* Güssow, Corynesporascaceae Sivan.). *Halojullela avicenniae* is a fungus isolated from intertidal mangrove wood of Queensland (Hyde 1992; Ariyawansa et al. 2013). Little information is available about the Halojulellaceae family, to which many saprobic and lichen-forming fungi belong (Ariyawansa et al. 2013). *Corynespora* genus has a widespread distribution and includes 89 species of saprobes, pathogens, and endophytes fungi, some from woody and herbaceous plants, others on nematodes, and human skin (Dixon et al. 2009). *Corynespora cassiicola* (Berk. and M.A. Curtis) C.T. Wei, the type species, is an important fungus causing target-spot on a wide host range in tropical and subtropical countries, especially *Hevea brasiliensis* (Willd. ex A. Juss.) Müll. Arg. in Sri Lanka and other countries (de Liyanage et al. 1986).

Both MUT 4407 and MUT 4323 (isolated from matte and rhizomes, respectively) cluster in Biatriosporaceae (Hyde et al. 2013). In particular, MUT 4407 clusters sister to a clade consisting of *Biatriospora marina* K.D. Hyde and Borse*, Biatriospora* sp. and *Nigrograna mackinnonii* (Borelli) Gruyter, Verkley and Crous; according to our analysis, it could be regarded a species of genus *Biatriospora* K.D. Hyde and Borse (*Biatriospora* sp*.*). MUT 4323 clusters with high support (BPP = 1; MLB = 100%) with *Massarina rubi* (Fuckel) Sacc. The genus *Biatriospora* was introduced by Hyde and Borse (1986) for *B. marina*, a mangrove inhabiting species. After that, it has been collected many times from a wide range of hosts showing a wide distribution (Hyde et al. 2013). *Nigrograna mackinnonii* is a species recently described from mycetomas of humans (de Gruyter et al. 2012). *Massarina* Sacc. is a polyphyletic genus (see below) of saprobic fungi that live both in terrestrial and aquatic environments (Fallah and Shearer 2001; Hyde et al. 2013). Moreover, *Massarina rubi* is a species sometimes recorded from freshwater (Fallah and Shearer 2001).

MUT 4397 and MUT 4419, both isolated from rhizomes, are clearly assigned to the Roussoellaceae family, under definition by JK Liu et al. (Hyde et al. 2013). Most of the taxa including in Roussoellaceae have been isolated from bamboo and palms (Hyde et al. 2013). The position of these two strains within this family is unclear and the molecular analyses did not allow including them in any of the known genera.

MUT 4405, isolated from rhizomes, groups together with *Massarina corticola* (Fuckel) L. Holm in an *incertae sedis* position close to Lophiostomataceae Sacc. as already signalled for *M. corticola* by Suetrong et al. (2009) (basal part of Clade XII – Lophiostomatceae); according to our LSU phylogenetic analyses, it could be considerate as *Massarina* sp. *Massarina corticola* was reported from submerged freshwater wood from China (Tsui et al. 2000, 2001). As said before, *Massarina* is a polyphyletic genus (Zhang et al. 2009); most of the marine *Massarina* species that have been sequenced have been referred to other families, while other species as *M. beaurivagea* Poonyth, K.D. Hyde, Aptroot and Peerally*, M. cystophorae* (Cribb and J.W. Herb.) Kohlm. and E. Kohlm., *M. lacertensis* Kohlm. and Volkm.-Kohlm., *M. mauritiana* Poonyth, K.D. Hyde and Aptroot*, M. rhizophorae* Poonyth, K.D. Hyde, Aptroot and Peerally and *M. ricifera* Kohlm., Volkm.-Kohlm. and O.E. Erikss. require study to confirm their position within this family (Jones et al. 2009).

### SM of *P. oceanica*in Capnodiales (Dothideomycetes)

The sequence belonging to the Capnodiales-Dothideomycetes clusters within the Mycosphaerellaceae Lindau. In Figure 2 is represented the Bayesian phylogram of Capnodiales taxa, including a sterile mycelium isolated from *P. oceanica* (Tables 1 and 2).

The Capnodiales are mainly defined by their shared ecological niche as leaf epiphytes associated with the honey dew produced by insects and saprobes or leaf pathogens and are an ascostromatal order without pseudoparaphyses (Hughes 1976; Crous et al. 2009a; Chomnunti et al. 2011).

MUT 4389, isolated from matte, has been identified as *Ramularia endophylla* Verkley and U. Braun according to the ITS and LSU sequence analysis (teleomorph *Mycosphaerella punctiformis* (Pers.) Starbäck (Crous 2009)). *Mycosphaerella punctiformis* (*Mycosphaerellaceae*), type species of the genus, is a plant pathogen belonging to a common genus in seawater (Verkley et al. 2004). Recent molecular studies have shown that *Mycosphaerella* Johanson is a polyphyletic genus (Crous et al. 2007, 2009b), with members belonging to many different genera, and even families, such as the Davidiellaceae C.L. Schoch, Spatafora, Crous and Shoemake*r*, Teratosphaeriaceae Crous and U. Braun, Dissoconiaceae Crous and de Hoog, Mycosphaerellaceae and Schizothyriaceae Höhn. ex Trotter, Sacc., D. Sacc. and Traverso. Members of the *Mycosphaerella-*complex are ecologically highly adaptable, and vary from being saprobic to fungicolous (Crous et al. 2009a, [b]). *Mycosphaerella* species are also among the most common and destructive known plant pathogens, causing serious diseases on many economically important crops. Species are mainly foliicolous, although some of them are associated with stem cankers, fruit lesions or blemishes, spots and specks (Crous et al. 2011).

On the whole, the majority of the Dothideomycetes analysed in this study has been isolated from *P. oceanica* matte, mainly constituted by decaying plant debris. This could explain why most of the SM can be ascribed to families (i.e. Cucurbitariaceae, Didymellaceae, Mycosphaerellaceae and Pleosporaceae,) that include numerous taxa that can play multiple ecological roles as saprotrophs or opportunistic pathogens (*Didymella, Phoma, Pyrenochaeta,* and *Stagonosporopsis* spp.).

### SM of *P. oceanica*in Letiomycetes

The six sequences belonging to the Leotiomycetes clustered in two subclades, *incertae sedis* and Helotiaceae 1 sensu Nekoduka et al*.* (2010), belonging both to two major clades here named Helotiales 1 and Helotiales 2, as shown by the Bayesian phylogram of Leotiomycetes taxa (Figure 3, Tables 1 and 2).

Leotiomycetes usually comprises plant-associated fungi whose ecologies range from pathogens (e.g. *Sclerotinia* Fuckel, *Blumeria* Golovin ex Speer), endophytes (e.g. *Phacidium* Fr.), saprobes (e.g. *Lachnum* Retz.), and mycorrhizal symbionts (e.g. *Hymenoscyphus* Gray) and a large number of taxa whose ecology and nutritional modes are poorly understood but are assumed to be plant based (Grünig and Sieber 2005; Wang *et al.*2006a, [b]). Members of Helotiales, the largest order in Leotiomycetes and one of the largest non lichen-forming ascomycetous groups, thrive in various ecosystems and cover a broad range of niches. The order was recently shown to be polyphyletic on molecular basis (e.g. Wang et al. 2006a, [b]; Nekoduka et al. 2010).

Whereas numerous leotiomycetous taxa are typical of freshwater habitats (Raja et al. 2008), only a few taxa (eg. *Amylocarpus* Curr., *Vibrissea* Fr., *Laetinaevia* Nannf.) have been reported from marine environments (Jones et al. 2009; Jones and Pang 2012b).

MUT 4415 and MUT 4416 (from rhizomes and matte, respectively) represent the same taxon (100% of ITS sequence identity), closely related with *Crocicreas cyathoideum* var*. cacaliae* (Pers.) S.E. Carp. on the basis of the ITS sequences (99% of sequence identity and 90% and 86% of query coverage, respectively with a reference GenBank sequence of *C. cyathoideum* var*. cacaliae,* see Table 2), and cluster with *Crocicreas amenti* (Batsch) S.E. Carp*.*, *C. coronatum* (Bull.) S.E. Carp. and *C. culmicula* (Desm.) S.E. Carp. in a clade consisting of *Cyathicula microspore* Velen*.*, *Ombrophila violacea* (Hedw.) Fr., *Hymenoscyphus scutula* (Pers.) W. Phillips*, Cudoniella clavus* (Alb. and Schwein.) Dennis on the basis of the LSU sequences (Helotiaceae 1 sensu Nekoduka et al. 2010 = the *Hymenoscyphus* clade sensu Raja et al. 2008 = the Cudoniella-Ombrophila clade sensu Wang et al. 2006b); these four genera are characterized by forming, especially in wet environments, small stipitate apothecioid ascomata on plant debris. *Crocicreas cyathoides* var. *cacaliae* is a rare species never found in the sea that generally grow as endophyte in stem of plants belonging to Fabaceae Lindl. family (Grau and Podlech 1996).

MUT 4401, MUT 4402, MUT 4412, from matte, and MUT 4411 from rhizomes, form a well-supported *incertae sedis* clade (BPP = 1, MLB = 95%) in the Helotiales 1, together with two sequences of *Mycochaetophora gentianae* Tak. Kobay., Kasuyama and Nasu, one sequence of *Mycochaetophora* sp., one sequence of *Rhexocercosporidium carotae* (Årsvoll) U. Braun and three sequences of *Cadophora* Lagerb. and Melin. The phylogenetic affinity among *Cadophora* spp., *Mycochaetophora gentianae* and *Rhexocercosporidium carotae* was previously pointed out (Nekoduka et al. 2010). *Mycochaetophora gentianae* is the causal agent of brown leaf spot on gentian (*Gentiana scabra* Bunge) (Nekoduka et al. 2013) whereas *Rhexocercosporidium carotae* causes blackish lesions on carrots during storage (Kastelein et al. 2007); *Cadophora* encompasses different saprotrophic and phytopatogenic species in terrestrial and aquatic environments (Harrington and Mcnew 2003; Burgaud et al. 2009; Goncalves et al. 2012). While MUT 4411 is classifiable as *R. carotae* (BPP = 0.99; MLB =71), MUT 4401 (identified as *Cadophora* sp. using the ITS analysis, see Table 2), MUT 4402 and MUT 4412 probably represent new taxa of *Cadophora,* and according to our phylogenetic analyses could represent new species within this genus.

Wang et al. (2006a) suggest that life style and ecological factors play a critical role in shaping the evolutionary history of helotialean fungi. All the species of *Cadophora*, *Rhexocercosporidium* and *Mycochaetophora* so far known share tolerance of cold environments, and they might constitute a cold-tolerant clade (Nekoduka et al. 2010).

Overall, the majority of Leotiomycetes found, mainly isolated from rhizomes (modified stems) of *P. oceanica,* clusters together with well-known phytopathogenic fungi. Noteworthy MUT 4415 and MUT 4416 cluster within the Helotiaceae clade together with taxa (ie *Crocicreas, Cudoniella* and *Hymenoscyphus* spp.) usually growing on dead submerged branches.

## Conclusions

The culture–independent molecular description of the microbial diversity from a number of natural habitats has revealed hitherto unknown microbial wealth, showing a new dimension of fungal diversity by bringing to light the presence of novel environmental phylotypes from a variety of marine habitats. However, in the absence of fungal isolates available in the culture collections, the correct identification (the systematic classification) of a microorganism is often impossible.

Six SM belong to genera firstly reported from marine environments. LSU marker analysis coupled to ITS data allowed to identify five SM at species level and nine to genus level. In the other cases it was not possible to go beyond the family or class level. Future molecular analyses, including additional gene sequences for increased resolution, could provide additional evidence for considering some of these as independent genera or phyletic lines.

The presented results contribute to the understanding of the marine fungal biodiversity, highlighting the systematic correlations of 21 sterile fungi isolated from *P. oceanica* meadows, a seriously threatened Mediterranean phytocenosis. These new information about their phylogenetic relationships suggest that they can play multiple ecological roles as saprotrophs or opportunistic pathogens in marine environments. The results clearly showed that all the sterile fungi belong to Dothideomycetes (Pleosporales and Capnodiales) and Leotiomycetes (Helotiales), despite the phylogenetic relationship of some of them remain to be determined since a majority of them may belong to new lineages of Ascomycota.

## Methods

### Fungal collections

The analysed SM have been isolated in a previous work (Panno et al. 2013), from matte and rhizomes of *P. oceanica* growing in meadow localized in Punta Manara close to Riva Trigoso Bay - Liguria, Italy (44° 15′ 00″ North - 9° 24′ 00″ West). A total of nine plants with the surrounding matte were collected in March 2008 at a depth between −5 and −21 m. Plants were placed in sealed sterile bags and maintained at about 4°C during transport. Within few hours the samples were serially washed with sterile water and divided into leaves, rhizomes, roots and matte. Five grams (fresh weight) of each composite sample were homogenized in 100 ml seawater sterilized by filtration (0.2 mm pores). The homogenates were diluted 1:10 using sterilized seawater and the final dilutions of each composite sample were plated (1 ml per plate) on different media prepared with seawater. More information about fungal isolation procedures is available in Panno et al. (2013).

In details, 21 out of the previously 29isolated SM were studied because the others were not able to survive in axenic conditions despite the use of different oligotrophic media prepared using seawater in order to mimic as much as possible the natural conditions.

All fungal strains were preserved at the *Mycotheca Universitatis Taurinensis*-MUT (DBIOS - University of Turin, Table 1).

### DNA extraction, PCR amplification and DNA sequencing

Genomic DNA was extracted using CetylTrimethyl Ammonium Bromide (CTAB, Sigma-Aldrich St. Louis, USA) following the protocol of Graham et al. (1994), and the nrDNA ITS1–5.8S–ITS2 and LSU partial regions were amplified using the universal primers ITS1F/ITS4 (Sigma-Aldrich St. Louis, USA) (White et al. 1990) and LR0R/LR7 (Vilgalys and Hester 1990 Vilgalys lab, unpubl., http://biology.duke.edu/fungi/mycolab/primers.htm), respectively. PCR amplifications were performed following parameters by White et al. (1990) for the ITS region, and Vilgalys and Hester (1990) for the LSU region. The analyses were carried out by sequencing the purified amplicons. Newly generated sequences were deposited in GenBank database (http://www.ncbi.nlm.nih.gov) and the accession numbers are shown in the Table 1.

### Bioinformatics and Phylogenetic analyses

The obtained sequences were checked and assembled using Geneious software (Drummond et al. 2010) and compared to those available in the GenBank database (http://www.ncbi.nlm.nih.gov) using the blastn algorithm and CBS Mycobank Pairwise Sequence Alignment (http://www.mycobank.org/). A full phylogenetic analysis was performed only on LSU/LSU-5.8S sequences, since comparable ITS sequences of fungi involved in the present paper were scant in public databases. Taxonomic assignment to SM based on ITS sequences was carried out by querying with the blastn algorithm the SM representative ITS sequence against the GenBank database (http://www.ncbi.nlm.nih.gov/Genbank/). For each isolated taxon the percentage ITS sequence identity value was provided (see Table 2). Blastn results were inspected manually to remove inconsistencies.

LSU sequences were selected for phylogenetic analysis on the basis of blastn and CBS (http://www.cbs.knaw.nl/) results. Accordingly, three sequences datasets were composed following Suetrong et al. (2009) and Hyde et al. (2013) for Pleosporales and Capnodiales Woron. (LSU datasets), and Wang et al. (2006a, [b]) and Nekoduka et al. (2010) for Leotiomycetes (LSU + 5.8S dataset). *Hysterium angustatum* Pers., *Hysterobrevium smilacis* (Schwein.) E.W.A. Boehm and C.L. Schoch and *Psiloglonium simulans* (W.R. Gerard) E.W.A. Boehm, C.L. Schoch and Spatafora (Hysteriales Lindau, Hysteriaceae Chevall.) were selected as outgroup taxa for the Pleosporales (Dothideomycetes) dataset, *Dothidea insculpta* Wallr., *Stylodothis puccinioides* (DC.) Arx and E. Müll. and *Dothiora cannabinae* Froid. (Dothideales, Dothideaceae), for the Capnodiales (Dothideomycetes) dataset, while *Peziza varia* (Hedw.) Alb. and Schwein. (Pezizomycetes O.E. Erikss. and Winka, Pezizales J. Schröt.) for the Leotiomycetes dataset.

Alignments were generated using MAFFT (Katoh et al. 2002) with default conditions for gap openings and gap extension penalties. The sequence alignments were refined manually with MEGA 5.10 (Tamura et al. 2011). Phylogenetic analyses were performed using Bayesian Inference (BI) and Maximum Likelihood (ML) approaches. The BI was performed with MrBayes 3.1.2 (Huelsenbeck and Ronquist 2001) with four incrementally heated simultaneous Monte Carlo Markov Chains (MCMC) run over 10 million generations, under GTR + Γ evolutionary model. Trees were sampled every 1,000 generations resulting in an overall sampling of 10,001 trees; the first 2,500 trees were discarded as “burn-in” (25%). For the remaining trees, a majority rule consensus tree showing all compatible partitions was computed to obtain estimates for Bayesian Posterior Probabilities (BPP). ML estimation was performed through RAxML v.7.3.2 (Stamatakis 2006) with 1,000 bootstrap replicates (Felsenstein 1985) using the GTRGAMMA algorithm to perform a tree inference and search for a good topology. Support values from bootstrapping runs (MLB) were mapped on the globally best tree using the “-f a” option of RAxML and “-x 12345” as a random seed to invoke the novel rapid bootstrapping algorithm. BI and ML analyses were run on the CIPRES Science Gateway web server (Miller et al. 2010). Only BPP values over 0.70 and MLB over 50 were reported in the resulting trees (Figures 1, 2 and 3).

Alignments and phylogenetic trees are available at TreeBASE (http://www.treebase.org*,* submission number S16179).

## References

[CR1] Abdel-Wahab MA, Bahkali AHA, Jones EBG, Pang KL (2012). Taxonomy of filamentous anamorphic marine fungi: morphology and molecular evidence. Marine fungi and fungal-like organisms, 1st edn.

[CR2] Alain K, Querellou J (2009). Cultivating the uncultured: limits, advances and future challenges. Extremophiles.

[CR3] Ariyawansa HA, Jones EBG, Suetrong S, Alias SA, Kang JC, Hyde KD (2013). Halojulellaceae a new family of the order Pleosporales. Phytotaxa.

[CR4] Aveskamp MM, de Gruyter J, Woudenberg JHC, Verkley GJM, Crous PW (2010). Highlights of the Didymellaceae: a polyphasic approach to characterise *Phoma* and related pleosporalean genera. Stud Mycol.

[CR5] Badali H, Chander J, Gulati N, Attri A, Chopra R, Najafzadeh MJ, Chhabra S, Meis JFGM, de Hoog GS (2010). Subcutaneous phaeohyphomycotic cyst caused by *Pyrenochaeta romeroi*. Med Biol.

[CR6] Baker PW, Kennedy J, Dobson DW, Marchesi JR (2008). Phylogenetic diversity and antimicrobial activities of fungi associated with *Haliclona simulans* isolated from Irish coastal waters. Mar Biotechnol.

[CR7] Burgaud G, Le Calvez T, Arzur D, Vandenkoornhuyse P, Barbier G (2009). Diversity of culturable marine filamentous fungi from deep-sea hydrothermal vents. Environ Microbiol.

[CR8] Chomnunti P, Schoch CL, Aguirre-Hudson B, Koko TW, Hongsanan S, Jones EBG, Kodsub R, Chukeatirote E, Bahkali AH, Hyde KD (2011). Capnodiaceae. Fungal Divers.

[CR9] Crous PW (2009). Taxonomy and phylogeny of the genus *Mycosphaerella* and its anamorphs. Fungal Divers.

[CR10] Crous PW, Braun U, Groenewald JZ (2007). *Mycosphaerella* is polyphyletic. Stud Mycol.

[CR11] Crous PW, Schoch CL, Hyde KD, Wood AR, Gueidan C, de Hoog GS, Groenewald JZ (2009). Phylogenetic lineages in the Capnodiales. Stud Mycol.

[CR12] Crous PW, Summerell BA, Carnegie AJ, Wingfield MJ, Hunter GC, Burgess TI, Andjic V, Barber PA, Groenewald JZ (2009). Unravelling *Mycosphaerella*: do you believe in genera?. Persoonia.

[CR13] Crous PW, Tanaka K, Summerell BA, Groenewald JZ (2011). Additions to the *Mycosphaerella* complex. IMA Fungus.

[CR14] Crous PW, Braun U, Hunter GC, Wingfield MJ, Verkley GJM, Shin HD, Nakashima C, Groenewald JZ (2013). Phylogenetic lineages in *Pseudocercospora*. Stud Mycol.

[CR15] Cuomo V, Vanzanella F, Fresi E, Cinelli F, Mazzella L (1985). Fungal flora of *Posidonia oceanica* and its ecological significance. T Brit Mycol Soc.

[CR16] Damare S, Raghukumar C, Raghukumar S (2006). Fungi in deep-sea sediments of the Central Indian Basin. Deep-Sea Res Pt I.

[CR17] de Gruyter JD, Woudenberg JH, Aveskamp MM, Verkley GJ, Groenewald JK, Crous PW (2010). Systematic reappraisal of species in *Phoma* section *Paraphoma*, *Pyrenochaeta* and *Pleurophoma*. Mycologia.

[CR18] de Gruyter J, Woudenberg JHC, Aveskamp MM, Verkley GJM, Groenewald JZ, Crous PW (2012). Redisposition of *Phoma*-like anamorphs in Pleosporales. Stud Mycol.

[CR19] de Hoog GS, Guarro J, Gené J, Figueras MJ (2000). Atlas of Clinical Fungi.

[CR20] de Liyanage AS, Jayasinghe CK, Liyanage NS, Jayaratne R (1986). *Corynespora* leaf spot disease of rubber (*Hevea brasiliensis*) a new record. J Rubber Res Institute of Sri Lanka.

[CR21] Dixon LJ, Schlub RL, Pernezny K, Datnoff LE (2009). Host specialization and phylogenetic diversity of *Corynespora cassiicola*. Phytopathology.

[CR22] Drummond AJ, Ashton B, Buxton S, Cheung M, Cooper A, Duran C, Field M, Heled J, Kearse M, Markowitz S, Moir R, Stones-Havas S, Sturrock S, Thierer T, Wilson A (2010). Geneious v5.3.

[CR23] Egidi E, de Hoog GS, Isola D, Onofri S, Quaedvlieg W, de Vries M, Verkley GJM, Stielow JB, Zucconi L, Selbmann L (2014). Phylogeny and taxonomy of meristematic rock-inhabiting black fungi in the Dothideomycetes based on multi-locus phylogenies. Fungal Divers.

[CR24] Fallah PM, Shearer CA (2001). Freshwater Ascomycetes: new or noteworthy species from north temperate lakes in Wisconsin. Mycologia.

[CR25] Felsenstein J (1985). Confidence limits on phylogenies: an approach using the bootstrap. Evolution.

[CR26] Garzoli L (2013). PhD Thesis. Marine Fungi in the Mediterranean Sea: Discovering their Diversity and their Potential Application in Biotechnology.

[CR27] Goncalves VN, Vaz A, Rosa CA, Rosa LH (2012). Diversity and distribution of fungal communities in lakes of Antarctica. FEMS Microbiol Ecol.

[CR28] Graham GC, Mayers P, Henry RJ (1994). A simplified method for the preparation of fungal genomic DNA for PCR and RAPD analysis. Biotechniques.

[CR29] Grau J, Podlech D (1996). Sendtnera.

[CR30] Grünig CR, Sieber TN (2005). Molecular and phenotypic description of the widespread root symbiont *Acephala applanata* gen. et sp. nov., formerly known as dark-septate endophyte Type 1. Mycologia.

[CR31] Harrington TC, McNew DL (2003). Phylogenetic analysis places the *Phialophora*-like anamorph genus *Cadophora* in the Helotiales. Mycotaxon.

[CR32] Huelsenbeck JP, Ronquist F (2001). MrBayes: Bayesian inference of phylogeny. Bioinformatics.

[CR33] Hughes SJ (1976). Sooty moulds. Mycologia.

[CR34] Hyde KD (1992). *Julella avicenniae* (Borse) comb. nov. (Thelenellaceae) from intertidal mangrove wood and miscellaneous fungi from the NE coast of Queensland. Mycol Res.

[CR35] Hyde KD, Borse BD (1986). Marine fungi from Seychelles V. *Biatriospora marina* gen. et sp. nov. from mangrove wood. Mycotaxon.

[CR36] Hyde KD, Jones EBG, Liu JK, Ariyawansa H, Boehm E, Boonmee S, Braun U, Chomnunti P, Crous PW, Dai DQ, Diederich P, Dissanayake A, Doilom M, Doveri F, Hongsanan S, Jayawardena R, Lawrey JD, Li YM, Liu YX, Lücking R, Monkai J, Muggia L, Nelsen MP, Pang KL, Phookamsak R, Senanayake IC, Shearer CA, Suetrong S, Tanaka K, Thambugala KM (2013). Families of Dothideomycetes. Fungal Divers.

[CR37] Imhoff JF, Labes A, Wiese J (2011). Bio-mining the microbial treasures of the ocean: new natural products. Biotechnol Adv.

[CR38] Jones EBG (1963). Marine fungi: II. Ascomycetes and deuteromycetes from submerged wood and drift Spartina. T Brit Mycol Soc.

[CR39] Jones EBG, Pang KL (2012). Marine fungi and fungal-like organisms.

[CR40] Jones EBG, Pang KL (2012). Tropical aquatic fungi. Biodivers Conserv.

[CR41] Jones EBG, Sakayaroj J, Suetrong S, Somrithipol S, Pang KL (2009). Classification of marine Ascomycota, anamorphic taxa and Basidiomycota. Fungal Divers.

[CR42] Kastelein P, Stilma ESC, Elderson J, Koehl J (2007). Occurrence of *Rhexocercosporidium carotae* on cold stored carrot roots in the Netherlands. Eur J Plant Pathol.

[CR43] Katoh K, Misawa K, Kuma K, Miyata T (2002). MAFFT: a novel method for rapid multiple sequence alignment based on fast Fourier transform. Nucleic Acids Res.

[CR44] Kirk PM, Cannon PF, Minter DW, Stalpers JA (2008). Ainsworth and Bisby’s Dictionary of the Fungi.

[CR45] Kohlmeyer J, Volkmann-Kohlmeyer B, Eriksson OE (1995). Fungi on *Juncus roemerianus* 2. New dictyosporous ascomycetes. Bot Mar.

[CR46] Mazzella L, Alberte RS (1986). Light adaptation and the role of autotrophic epiphytes in primary production of the temperate seagrass, *Zostera marina* L. J Exp Mar Biol Ecol.

[CR47] Meyers SP, Orpurt PA, Simms J, Boral LL (1965). Thalassiomycetes VII. Observations on fungal infestation of turtle grass, *Thalassia testudinum* König. B Mar Sci.

[CR48] Miller MA, Pfeiffer W, Schwartz T (2010). Creating the CIPRES Science Gateway for inference of large phylogenetic trees. IEEE.

[CR49] Morrison-Gardiner S (2002). Dominant fungi from Australian coral reefs. Fungal Divers.

[CR50] Nekoduka S, Tanaka K, Harada Y, Sano T (2010). Phylogenetic affinity of *Mycochaetophora gentianae*, the causal fungus of brown leaf spot on gentian (*Gentiana triflora*) to *Pseudocercosporella*-like hyphomycetes in Helotiales. Mycoscience.

[CR51] Nekoduka S, Tanaka K, Sano T (2013). Overwintering of brown leaf spot fungus, *Mycochaetophora gentianae*, in infected gentian leaves as the primary inoculum source. J Gen Plant Pathol.

[CR52] Newell SY (1981). Fungi and bacteria in or on leaves of eelgrass (*Zostera marina* L.) from Chesapeake Bay. Appl Environ Microb.

[CR53] Panno L, Bruno M, Voyron S, Anastasi A, Gnavi G, Miserere L, Varese GC (2013). Diversity, ecological role and potential biotechnological applications of marine fungi associated to the seagrass *Posidonia oceanica*. New Biotechnol.

[CR54] Perez-Ortega S, Suija A, Crespo A, de los Rios A (2014). Lichenicolous fungi of the genus *Abrothallus* (Dothideomycetes: Abrothallales ordo nov.) are sister to the predominantly aquatic Janhulales. Fungal Divers.

[CR55] Pergent G, Boudouresque CF, Dumay O, Pergent-Martini C, Wyllie-Echeverria S (2008). Competition between the invasive macrophyte *Caulerpa taxifolia* and the seagrass *Posidonia oceanica*: contrasting strategies. BMC Ecol.

[CR56] Prasannarai K, Sridhar KR (2001). Diversity and abundance of higher marine fungi on woody substrates along the west coast of India. Curr Sci India.

[CR57] Quaedvlieg W, Verkley GJM, Shin HD, Barreto RW, Alfenas AC, Swart WJ, Groenewald JZ, Crous PW (2013). Sizing up Septoria. Stud Mycol.

[CR58] Raghukumar S, Ramaiah N (2004). The role of fungi in marine detrital processes. Marine microbiology: facets and opportunities.

[CR59] Raja HA, Miller AN, Shearer CA (2008). Freshwater ascomycetes: *Aquapoterium pinicola*, a new genus and species of Helotiales (Leotiomycetes) from Florida. Mycologia.

[CR60] Rateb ME, Ebel R (2011). Secondary metabolites of fungi from marine habitats. Nat Prod Rep.

[CR61] Schoch CL, Crous PW, Groenewald JZ, Boehm EWA, Burgess TI, de Gruyter J, de Hoog GS, Dixon LJ, Grube M, Gueidan C, Harada Y, Hatakeyama S, Hirayama K, Hosoya T, Huhndorf SM, Hyde KD, Jones EBG, Kohlmeyer J, Kruys A, Li YM, Lücking R, Lumbsch HT, Marvanová L, Mbatchou JS, McVay AH, Miller AN, Mugambi GK, Muggia L, Nelsen MP, Nelson P (2009). A class-wide phylogenetic assessment of Dothideomycetes. Stud Mycol.

[CR62] Shearer CA, Raja HA, Miller AN, Nelson P, Tanaka K, Hirayama K, Marvanová L, Hyde KD, Zhang Y (2009). The molecular phylogeny of freshwater Dothideomycetes. Stud Mycol.

[CR63] Shoemaker RA, Babcock CE (1992). Applanodictyosporous Pleosporales: *Clathrospora, Comoclathris, Graphyllium, Macrospora,* and *Platysporoides*. Can J Botany.

[CR64] Stamatakis A (2006). RAxML-VI-HPC: maximum likelihood-based phylogenetic analyses with thousands of taxa and mixed models. Bioinformatics.

[CR65] Suetrong S, Schoch CL, Spatafora JW, Kohlmeyer J, Volkmann-Kohlmeyer B, Sakayaroj J, Phongpaichit S, Tanaka K, Hirayama K, Jones EBG (2009). Molecular systematics of the marine Dothideomycetes. Stud Mycol.

[CR66] Suryanarayanan TS (2012). The diversity and importance of fungi associated with marine sponges. Bot Mar.

[CR67] Tamura K, Peterson D, Peterson N, Stecher G, Nei M, Kumar S (2011). MEGA5: molecular evolutionary genetics analysis using maximum likelihood, evolutionary distance, and maximum parsimony methods. Mol Biol Evol.

[CR68] Toledo-Hernández C, Bones-González A, Ortiz-Vázquez OE, Sabat AM, Bayman P (2007). Fungi in the sea fan *Gorgonia ventalina*: diversity and sampling strategies. Coral Reefs.

[CR69] Tsui CKM, Hyde KD, Hodgkiss IJ (2000). Biodiversity of fungi on submerged wood in Hong Kong streams. Aquat Microb Ecol.

[CR70] Tsui CKM, Hyde KD, Hodgkiss IJ (2001). Longitudinal and temporal distribution of freshwater ascomycetes and dematiaceous hyphomycetes on submerged wood in the Lam Tsuen River, Hong Kong. J N Am Benthol Soc.

[CR71] Verkley JM, Crous PW, Groenewald JZ, Braun U, Aptroot A (2004). *Mycosphaerella punctiformis* revisited: morphology, phylogeny, and epitypification of the type species of the genus *Mycosphaerella*. Mycol Res.

[CR72] Vijaykrishna D, Jeewon R, Hyde KD (2006). Molecular taxonomy, origins and evolution of freshwater Ascomycetes. Fungal Divers.

[CR73] Vilgalys R, Hester M (1990). Rapid genetic identification and mapping of enzymatically amplified ribosomal DNA from several *Cryptococcus* species. J Bacteriol.

[CR74] Wang Z, Binder M, Schoch CL, Johnston PR, Spatafora JW, Hibbett DS (2006). Evolution of helotialean fungi (Leotiomycetes, Pezizomycotina): a nuclear rDNA phylogeny. Mol Phylogenet Evol.

[CR75] Wang Z, Johnston PR, Takamatsu S, Spatafora JW, Hibbett DS (2006). Toward a phylogenetic classification of the Leotiomycetes based on rDNA data. Mycologia.

[CR76] White TJ, Bruns T, Lee S, Taylor JW, Innis MA, Gelfand DH, Sninsky JJ, White TJ (1990). Amplification and direct sequencing of fungal ribosomal RNA genes for phylogenetics.

[CR77] Zhang Y, Crous PW, Schoch CL, Hyde KD (2012). Pleosporales. Fungal Divers.

[CR78] Zhang Y, Schoch CL, Fournier J, Crous PW, De Gruyter J, Woudenberg JHC, Hirayama K, Tanaka K, Pointing SB, Spatafora JW, Hyde KD (2009). Multi-locus phylogeny of the Pleosporales: a taxonomic, ecological and evolutionary re-evaluation. Stud Mycol.

[CR79] Zuccaro A, Conrad L, Spatafora LW, Kohlmeyer J, Draeger S, Mitchell JI (2008). Detection and identification of fungi intimately associated with the brown seaweed *Fucus serratus*. Appl Environ Microb.

